# Telehealth regulating together pilot trial: emotion regulation intervention for autistic children and adolescents

**DOI:** 10.3389/fpsyt.2024.1401148

**Published:** 2024-10-07

**Authors:** Marika Coffman, Miranda Wells, Lauren M. Schmitt, Debra L. Reisinger, Paul S. Horn, Rebecca C. Shaffer

**Affiliations:** ^1^ Department of Psychiatry and Behavioral Sciences, Duke Center for Autism and Brain Development, Duke University, Durham, NC, United States; ^2^ Department of Psychiatric and Behavioral Sciences, Duke University, Durham, NC, United States; ^3^ Division of Behavioral Medicine and Clinical Psychology, Cincinnati Children’s Hospital Medical Center, Cincinnati, OH, United States; ^4^ Department of Pediatrics, University of Cincinnati College of Medicine, Cincinnati, OH, United States; ^5^ Neurology Division, Cincinnati Children’s Hospital Medical Center, Cincinnati, OH, United States

**Keywords:** autism, emotion regulation, children, adolescents, telehealth, emotion dysregulation, group therapy, teletherapy

## Abstract

**Introduction:**

Autistic children and adolescents frequently experience emotion dysregulation, or difficulties with appropriately modifying their emotional reactions. Caregivers of autistic teens frequently seek psychotherapy support for navigating challenges associated with emotion dysregulation. During the COVID-19 pandemic, access to clinical services became limited, with interventions halted or transitioned into a telehealth format.

**Methods:**

This study evaluates the feasibility, acceptability, and initial efficacy of a telehealth adaptation to an existing intervention for emotion dysregulation for children and teens with autism, Regulating Together. A within-subjects trial was conducted for Child (ages 8-12) and Teen groups (ages 13-18). The trial consisted of a 5-week-control lead-in period, a 5-week-intervention, and 5-and 10-weeks-post-intervention follow-ups.

**Results:**

Twenty-eight youth with ASD + ED (n=13 Child and n=15 Teens, 71% male) participated. We observed a 93% retention rate across both groups. Improvements were found in reactivity, irritability, emotion and behavioral regulation, and flexibility immediately post-intervention and 10-weeks post-intervention in both groups. Additional improvements in dysphoria, cognitive regulation, and emotional control were observed in teens.

**Discussion:**

Our results suggest promising improvements in ED through telehealth delivery of an emotion regulation intervention in autistic children and adolescents, along with possible improvements in accessibility of this intervention.

## Introduction

Autistic children and adolescents frequently engage in interventions for behavioral challenges, such as tantrums, self- and other-directed aggression (1) and emotional difficulties, such as anxiety and depression. The COVID-19 pandemic impacted children’s and adolescent’s mental and behavioral health in a number of ways, including increased depression and anxiety, regardless of pre-existing psychiatric diagnoses (2). In addition, behavioral therapies during this time were often halted thus leaving these behavioral challenges unaddressed or delivered via telehealth, which at the time had not been thoroughly assessed for efficacy. Therefore, examining the feasibility, acceptability, and initial efficacy of emotion regulation therapies delivered via telehealth during this the COVID-19 pandemic may provide useful information for the continued use of telehealth as a means of improving emotion regulation in autistic children and adolescents.

Emotion dysregulation (ED) refers to the difficulties with modulating a response to expressing emotions in a contextually appropriate manner (3). ED can be characterized by increased reactivity, irritability, and challenges with calming oneself after becoming upset. ED is often observed in many psychiatric conditions like anxiety disorders (4) and depression (5). It has been hypothesized that the rigidity and emotional intensity that occurs in ED could be a contributor to the difficulties that autistic individuals face, such as disrupted social interactions and opportunities (3).

The COVID-19 pandemic and nation-wide lockdown disrupted everyone’s lifestyle in a variety of ways. These challenges disproportionally affected children with ASD, with one study finding up to 59% of autistic children (age 2-1 *M*=9.12) showed worsened psychiatric problems or new psychiatric diagnosis according to parent report in May of 2020 (6). For many families, interventions and therapies and stable social environments like school and extracurricular activities were cancelled. These disruptions could have potential long-term effects on a child’s development (7).

As a result of the COVID-19 pandemic, healthcare has adapted to include more telehealth options. These opportunities not only benefit individuals who cannot attend in-person appointments due to health concerns, but they also make healthcare more accessible, such as to those in rural areas where local resources are limited (8). Evidence suggests that the transition to telehealth at this time improved patient attendance and resulted in improvement in mental health symptoms in a predominantly low-income and racially diverse population (9). Similar success has been observed in telehealth for rural families of autistic children. In a study of a parent-mediated intervention (COMPASS for Hope) administered in a telehealth setting for families in rural and urban areas, the researchers found higher parent competence was associated with fewer child behavioral problems (10). This was similar to the results of the same intervention in face-to-face environments (10). This suggests that this therapy and possibly others could be effective across platforms (10).

In order to address the need for autism specific ED interventions, Shaffer et al. (11) developed Regulating Together (RT), an outpatient intervention training both autistic individuals and their caregivers on emotion regulation strategies. The curriculum was developed for two development age groups, children (8-12 years old) and teens (13-18 years old). In the pilot within subjects trial, RT was found to improve reactivity, emotion regulation knowledge, and flexibility across both age groups both immediately post intervention and up to 10 weeks after the intervention concluded (11). However, due to the onset of the COVID-19 pandemic, ongoing trials for RT transitioned to a telehealth format.

The current study aims to evaluate the feasibility, acceptability, and initial efficacy of RT in a telehealth modality for improving emotion regulation in autistic youths. Due to the previous success of RT and the promising results of telehealth interventions for individuals with ASD, we hypothesize that the telehealth delivery of RT will improve emotion regulation, as seen by increased flexibility as well as decreased reactivity, emotional dysphoria, and irritability. We also hypothesize that participants would show overall improvements in youth functioning according to the caregiver report.

## Methods

### Participants

Twenty-eight autistic participants between the ages of 8-18 years participated in an intervention study which utilized a within-subjects design. The intervention enrollment consisted of two Child groups (n=13) and two Teen groups (n=15). Demographics are presented in **Table 1**.

**Table 1:** Participant demographics.

	Younger group (N=13)		Older Group (N=15)		Total (N=28)	
Age	Mean (SD)	Range	Mean (SD)	Range	Mean (SD)	Range
	10.32 (1.51)	(8.06 - 12.38)	15.62 (1.54)	13.14 - 18.32	13.11 (3.06)	8.06-18.32
Vineland	Mean (SD)	Range	Mean (SD)	Range	Mean (SD)	Range
Communication Skills	83.54 (15.29)	68-92	77.36 (6.05)	57-105	80.33 (11.67)	57-105
Daily Living Skills	72.08 (20.48)	65-106	83.57 (10.09)	20-98	78.04 (16.70)	20-106
Socialization Skills	71.15 (14.49)	58-89	73.43 (8.89)	42-96	72.33 (11.74)	42-96
Adaptive Behavior Composite	75.92 (11.19)	65-92	75.29 (6.40)	53-96	75.59 (8.85)	53-96
Sex	Number	Percent	Number	Percent	Number	Percent
Male	8	61.54	12	80.00	20	71.43
Female	5	38.46	3	20.00	8	28.57
Race	Number	Percent	Number	Percent	Number	Percent
White	12	92.31	14	93.33	26	92.86
Black/African American	0.00	0	0.00	0	0.00	0
Asian	0.00	0	1	6.67	1	3.57
American Indian/Alaskan Native	1	7.69	0.00	0	1	3.57
Native Hawaiian*/*Pacific Islander	0.00	0	0.00	0	0.00	0
Other	0.00	0	0.00	0	0.00	0
Ethnicity*	Number	Percent	Number	Percent	Number	Percent
Hispanic	1	7.69	0.00	0	1	3.57
non-Hispanic	12	92.31	14	100.00	26	92.86
Co-occurring Psychiatric Diagnoses **	Percent	Number	Percent	Number	Percent	Number
ADHD	5	38.46	7	46.67	12	42.86
Anxiety*/*Panic Attacks	4	30.77	6	40.00	10	35.71
Bipolar Disorder	0.00	0	0.00	0	0.00	0
Depression	1	7.69	2	13.33	3	10.71
Intermittent Explosive Disorder	2	15.38	2	13.33	4	14.29
Insomnia	4	30.77	0.00	0	4	14.29
Obsessive Compulsive Disorder	0.00	0	3	20.00	3	10.71
Oppositional Defiant Disorder	1	7.69	0.00	0	1	3.57
Psychotic Disorder	0.00	0	0.00	0	0.00	0
Post-traumatic Stress Disorder	0.00	0	0.00	0	0.00	0
Schizophrenia	0.00	0	0.00	0	0.00	0
Substance Abuse	0.00	0	0.00	0	0.00	0
Other	4	30.77	4	26.67	8	28.57
Parent Highest Education Level	Number	Percent	Number	Percent	Number	Percent
High School or GED	1	7.69	2	13.33	3	10.71
Some College or 2-year degree	0.00	0	3	20.00	3	10.71
College Graduate	7	53.85	4	26.67	11	39.29
Advanced Graduate or Professional Degree	5	38.46	6	40.00	11	39.29

*One participant did not respond to ethnicity.

**Fifteen participants did not respond. The data shown reflects those who have a diagnosis of the psychiatric disorder.

Participants were recruited from multiple sources, including internal hospital clinics and community partners (e.g., schools, local autism agencies). Due to the telehealth format of the intervention, youth were recruited nationally through sharing the flyer with autism clinics outside our hospital. Consistent with prior RT groups (11), youth were eligible for study if they were between the ages 8–18 years, had a diagnosis of autism documented by a provider, had an IQ > 65, had their primary spoken language as English and spoke with functional verbal communication, commensurate with appropriateness to receive an ADOS-2 Module 3 or 4 (12), were willing to take a break from or keep other interventions stable, keep medication stable, and have at least one caregiver willing to participate in the intervention. A diagnosis of autism was confirmed through a medical and behavioral history interview and expert clinical diagnosis. Due to the COVID-19 pandemic, adaptations to the inclusion criteria included: participants who had any cognitive testing and/or an ADOS-2 completed within the past year and met diagnostic criteria for autism. Emotion dysregulation inclusion criteria were based on prior scores established by our group (20) the Aberrant Behavior Checklist, Second Edition (ABC-2; (13) was used, such that youth were eligible if a) they had scores of ≥ 10 on the irritability or hyperactivity subscales. To ensure safety for all study participants, any aggression toward other youth that resulted in injury in the past 2 weeks resulted in exclusion from the study. Of the children screened, there were ultimately two children who did not meet the inclusion criteria, one from the child group due to low ABC score and one from the teen group due to low IQ score (not included in the enrollment numbers above).

### Intervention

The intervention study protocol followed prior RT groups (11), such that all study participants completed a 5-week control lead-in period (T1-T5), a 5-week active intervention period (T5-T10) and follow up assessments at 5-weeks (T15) and 10-weeks (T20) post intervention completion. IRB approval was obtained from Cincinnati Children’s Hospital Medical Center, and all participants provided consent or assent, with caregivers providing consent for all participant younger than 18 years. Families received compensation commensurate with the time commitment for each study visit. The groups occurred after school in the early evening (between 4-6:30 EST), although it is important to note that youth were recruited from across the United States and resided in different time zones. Families adjusted their personal schedules to attend the groups as needed. The Child groups occurred in 2020 from June-July for the first round and October-November for the second round. Teen groups occurred in August-September 2020 and February-March 2021. The majority of youth across the second round of child and both teen rounds were back attending in person school at the time of the study completion.

The telehealth RT program was offered in two different formats depending on the child’s age. For the Child group, caregivers participated in a live, telehealth group sessions receiving the caregiver material twice weekly for 80 minutes across 5 weeks for a total of 10 sessions. They then facilitated recorded videos of the intervention one-on-one with their children before the next session. The video recordings included PowerPoint presentations with voice over by a psychologist, teaching the material in the same way as in person, with pauses for the caregivers to complete worksheets or discuss the topic further with their child. Each video took between 35-60 minutes for families to complete depending on how much discussion they had or how long the activities took the child to complete. Children and caregivers received workbooks in the mail before the start of the group with all the materials they needed over the course of the curriculum. The videos were posted after each caregiver group on the Play Posit platform, which tracked completion of viewing of the video. The children joined the last 10-15 minutes of each group to practice relaxation and briefly review the material. The Teen version included 60-minute live, telehealth caregiver group sessions followed by 60-minute live, telehealth adolescent group sessions. Caregivers were nearby during live adolescent sessions when needed to help ensure attention, but privacy was maintained as much as possible. They met twice weekly for 5 weeks (10 sessions). Teens and their caregivers received workbooks in the mail prior to the start of group with all materials included for the curriculum. PowerPoint presentations were used by the leader to help the teens focus and process the material and to match the in-person format, which delivers the intervention verbally with paired visuals. There was one psychologist who led both the caregiver and teen groups. A graduate student served as an assistant in the Teen groups. Details on the intervention components of RT are available in Shaffer et al. (11). Differences between the in-person and telehealth modalities are listed in **Table 2**.

**Table 2:** Differences between regulating together modalities.

Concept	Regulating Together (In-person)	Telehealth - Regulating Together	
		Child	Teen
**Caregiver Involvement**	Child/Teen material presented in group sessions, while caregivers attended simultaneous group training	Caregiver material presented live. Child material prerecorded and facilitated by caregiver	Caregiver and teen material presented live at separate times. Caregivers remained close by to ensure teen attention
**Timing**	90-minute sessions	80-minute Caregiver sessions (where child joined for the last 10-15 minutes) 35-60 minute Caregiver Facilitated Videos	60-minute Caregiver and 60-minute teen session
**Worksheets**	Worksheets provided in-person	Both groups received workbooks in the mail	
**Visual Materials**	Visuals through posters in group room	Visuals presented in PowerPoint	
**Clinician Involvement**	Two Doctoral or Masters Level Clinicians (One leading Child/Teen group, one leading Caregiver group)	Same psychologist leading both groups	
**Staff Involvement**	Behavior Assistant in Child/Teen groups	No Behavioral Tech support in home A graduate student provided assistance in the Teen group	
**Learning Materials**	All the Same Material Covered		

#### Feasibility and acceptability

Feasibility was measured via attendance and retention across the study. Acceptability was measured via youth self-ratings of how much they learned and how they felt in each session. Self-ratings of learning were scored on a 5-point Likert scale from 1 = Nothing to 5 = Very Much. Self-ratings of feelings were also measures on a 5-point Likert scale from 1 = Calm and in Control to 5 = Out of Control. Self-ratings of feelings were completed after the second session for teens and fourth session for children, concordant with in session teaching of the rating scale.

#### Caregiver satisfaction

Caregivers were asked to report their satisfaction with the intervention by answering five questions on a 5-point Likert scale anchors for each are included by each question. These questions asked caregivers about how much they learned (5 = Learned a great deal, 1 = Learned nothing), how satisfied they were with the material (5 = Very Satisfied, 1 = Very Dissatisfied), how confident they feel in managing their child’s behaviors (5 = Very Confident, 1 = Very Unconfident), how well they can implement the skills (5 = Very Well, 1 = Not at all), and how well their child is implementing the skills taught in the group (5 = Very Well, 1 = Not at all).

### Measures

A comprehensive assessment battery was collected to examine the efficacy of RT delivered through telehealth. Demographics of study participants and their families were collected through interviews and surveys. All measures were used in the primary RT evaluation (11) and are described below. The measures were completed during a telehealth meeting with the study coordinator or clinician on screen with the family to provide instructions, answer questions, and make sure the same respondent completed the measures each time. Links were sent to families ahead of the appointment so they could complete the measures on their own screen. If they did not have access to a screen and struggled with completion on their phone, the coordinator could read the questions and mark answers for the family. This was a rare occurrence and the majority of families completed measures on their own screens.

#### Vineland scales of adaptive behavior, third edition (Vineland-3)

The Vineland Scales of Adaptive Behavior, Third Edition (Vineland-3) is a standardized measure of adaptive behavior that assesses skills across the following domains: Communication, Daily Living Skills, and Socialization domains. It also provides an overall Adaptive Behavior Composite score. The Vineland-3 was used at screening to characterize the overall functioning of participants. Caregivers completed the Parent form.

### Primary and secondary outcome measures

#### The aberrant behavior checklist - irritability

The Aberrant Behavior Checklist [ABC-2 (13)] is a caregiver-report questionnaire comprised of 58 items. There are five subscales of the ABC-2: Irritability, Social Withdrawal/Lethargy, Stereotypy, Hyperactivity, and Inappropriate Speech. Caregivers rate the severity of behaviors on a 4-point Likert scale ranging from 0 = Not a Problem to 3 = the Problem is Severe in degree. The ABC-2 was collected for both study inclusion and a primary study outcome.

#### The emotion dysregulation inventory

The Emotion Dysregulation Inventory [EDI (14)] is a caregiver report questionnaire that consists of two scales: Reactivity (EDI-R), and Dysphoria (EDI-D). The EDI-R subscale was collected to serve as the primary outcome measure for this study.

#### The clinical global impressions scale-improvement

The Clinician Global Impressions - Improvement [CGI-I; (15)] is a clinician-rated measure that assesses response to intervention. This measure was completed by an independent and trained clinician at T5, T10, T15 and T20. The CGI-I is scored on a 7-point scale ranging from 1 = Very Much Improved to 7 = Very Much Worse. CGI-S, but not CGI-I, was completed at T1. CGI-I was not completed at T1 due to it being an improvement measure based on ratings and clinical information gathered for CGI-S. Consistent with our published pilot of RT in person (11), the CGI-I clinician (LS) was a psychologist who completed reliability training with gold standard vignettes. She was not involved in the treatment and was aware that this was a within-subjects trial. The CGI-I rating was completed per standard administration with a parent interview regarding global changes observed in different environments with specific emphasis on changes in emotion dysregulation (e.g., changes in severity, duration, and frequency of outbursts; changes in handling triggers).

#### Flexibility scale

The Flexibility Scale [FS (16)] is a caregiver report measure that assesses flexibility in autistic youth. Caregivers score responses on a 4-point Likert scale ranging from 0 = No to 3 = Always. This measure was collected as a secondary outcome measure. Subscales on this measure include: Social Flexibility, Transitions/Change, Generativity, and Total.

#### Behavioral rating inventory of executive functioning

The Behavioral Rating Inventory of Executive Functioning [BRIEF-2 (17)] is caregiver-report measure consisting of 86 items. Caregivers rate their child’s behavior on a 3-point Likert scale ranging from 1 = Never to 3 = Often. Raw scores are then converted to T-scores for the individual scales and standard scores for the Indexes, which include: Behavior Regulation Index (BRI), Cognitive Regulation Index (CRI), Emotional Regulation Index (ERI), and an overall Global Executive Composite (GEC). The ERI was selected as a secondary outcome measure for the present study.

### Statistical analysis

#### Primary analysis

Repeated measures ANOVA was utilized to analyze our outcome variables involving data from the 5-week control period (T1-T5), through the intervention period (T5-T10), and the final post intervention outcome period, (T5-T20). Outcome measures included the EDI-R and EDI-D, ABC-2, BRIEF-2, and the Flexibility Scale. All participants (Child & Teen) were analyzed together for the primary analyses. Due to the pilot nature of this study, corrections for multiple comparisons were not conducted. Generalized linear mixed models were examined with various distributions and within subject covariance structures. The models chosen exhibited reasonable fit using the corrected Akaike Information Criterion as well as displaying good profiles of the studentized residuals.

#### Secondary analysis

Individual timepoints were examined as a secondary analysis.

#### Demographic analyses

Means and standard deviations were calculated for demographic data including: age, gender, parental education, race, ethnicity, and co-occurring psychiatric diagnoses.

## Results

### Feasibility

Out of all participants enrolled, one child dropped out of the study during the control lead-in period and one teen dropped during the intervention period due to difficulty paying attention in the telehealth format. We thus demonstrate a retention rate of 93% over the course of the entire study for both the Child and Teen groups. For the intervention phase specifically, there was a 96% retention rate. For the Child group, caregiver attendance to the live sessions was 94% and completion of the videos for the children (as measured in Play Posit platform) was 98%. For the Teen group, there was a 94% attendance rate for caregivers and teens. Compared to our in-person RT data, telehealth RT had better retention (93%>87%) and attendance (94%>82%). The telehealth format appeared to be more feasible and accessible for families compared to in person attendance, even when in person feasibility was acceptable.

### Acceptability

**Figure 1:**
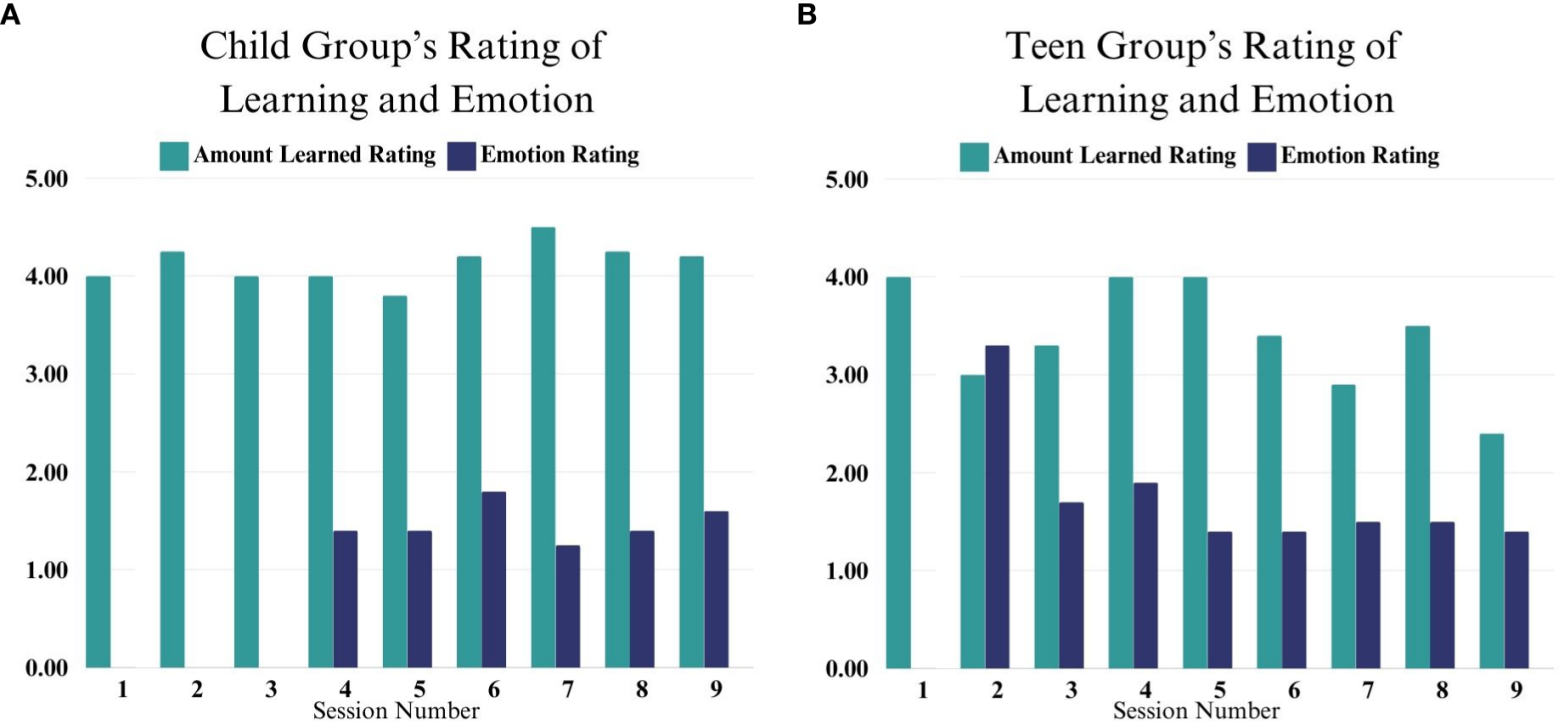
**(A, B)** These figures depict Child and Teen self-rating of their learning and emotion across sessions 1-9. Emotion ratings began in Session 4 for the Child Group and Session 2 for the Teen Group.

In the Child group, participants rated that they learned the most in Sessions 7 (Positive and Negative Thoughts, M = 4.5), 2 (Relaxation, M = 4.2), and 8 (Cognitive Flexibility, M = 4.25). Overall, the children rated themselves calm at the end of viewing the videos (M = 1.25-1.8) (as shown in **Figure 1A**). For the Teens, they rated that they learned the most in Session 1 (Relaxation, M= 4), Session 4 (Distress Tolerance, M = 4), and Session 5 (Problem Sizes & Positive Thoughts, M = 4). Teens similarly rated themselves as having low levels of arousal at the end of each group (M = 1.4-1.9) with the exception of Session 2 when they learned the 5-point scale for the first time (as shown in **Figure 1B**). Several teens rated themselves as a 5, which was not consistent with their presentation in group, and it is possible they misunderstood the direction of the scale. For all future sessions, the anchors of the scale were clearly labeled at the bottom.

### Caregiver satisfaction

Immediately following the group, caregivers reported high levels of satisfaction with the telehealth format of RT. In particular, caregivers reported that they were satisfied with the group (M = 4.65, SD = 0.49), that they learned skills from the group (M = 4.38, SD = 0.57), that they feel confident managing their child’s behavior (M = 3.69, SD = 1.01), and that they think they are implementing the skills well (M = 3.58, SD = 0.76). Parents reported the least amount of confidence in their child implementing the skills (M = 2.96, SD = 0.66).

### Adverse events

There were no reported behavior changes for youth in the Child or Teen samples and there were no inpatient hospitalizations over the course of the study for participants. There were no reported instances of emotional outbursts during the telehealth sessions.

### Primary outcome measures

Results comparing individual time periods by age groups are presented in **Table 3**. Results from each age group separately are presented in **Table 4**.

**Table 3:** Mixed model analysis by time period.

Measure	Younger			Older		
	t	p	Cohen’s d	t	p	Cohen’s d
Emotion Dysregulation Inventory						
Emotion Dysregulation Inventory – Reactivity (Theta)						
**T1-T5**	0.20	0.846	0.05	0.06	0.950	0.01
**T1-T10**	**2.54**	**0.014**	0.90	**2.37**	**0.022**	0.45
**T1-T15**	**2.83**	**0.007**	1.03	1.78	0.083	0.38
**T1-T20**	**3.05**	**0.004**	1.17	**3.92**	**<0.001**	0.81
**T5-T10**	**2.76**	**0.009**	0.86	**2.36**	**0.023**	0.45
**T5- T15**	**2.79**	**0.007**	0.99	1.73	0.092	0.38
**T5-T20**	**2.93**	**0.005**	1.13	**3.86**	**<0.001**	0.84
**T10-T15**	0.62	0.541	0.18	-0.47	0.640	-0.14
**T10-T20**	0.79	0.434	0.37	1.60	0.118	0.36
**T15-T20**	0.30	0.767	0.18	**2.03**	**0.048**	0.57
Emotion Dysregulation Inventory – Dysphoria (Theta)						
***Theta***						
**T1-T5**	0.35	0.730	-0.39	-0.88	0.385	0.47
**T1-T10**	2.44	0.018	0.51	1.97	0.055	1.28
**T1-T15**	1.66	0.103	0.62	**2.07**	**0.048**	0.74
**T1-T20**	1.74	0.088	0.64	**3.09**	**0.006**	1.09
**T5-T10**	2.65	0.011	0.97	**3.00**	**0.004**	0.78
**T5- T15**	1.54	0.129	1.12	**2.80**	**0.008**	0.24
**T5-T20**	1.58	0.120	1.10	**3.78**	**0.001**	0.55
**T10-T15**	-0.71	0.483	0.10	0.31	0.761	-0.57
**T10-T20**	-0.36	0.720	0.16	1.31	0.199	-0.30
**T15-T20**	0.25	0.801	0.07	1.13	0.265	0.31
Aberrant Behavior Checklist - Irritability Subscale						
**T1-T5**	0.90	0.374	0.28	**2.73**	**0.009**	0.47
**T1-T10**	**3.21**	**0.003**	0.95	**4.23**	**<0.001**	0.75
**T1-T15**	**3.24**	**0.003**	1.10	**3.57**	**0.002**	0.75
**T1-T20**	**3.67**	**0.002**	1.44	**4.27**	**<0.001**	1.15
**T5-T10**	**3.20**	**0.003**	0.70	**2.71**	**0.009**	0.41
**T5- T15**	**2.95**	**0.006**	0.85	2.01	0.051	0.36
**T5-T20**	**3.33**	**0.003**	1.20	**2.86**	**0.010**	0.79
**T10-T15**	0.64	0.524	0.13	-0.07	0.947	-0.09
**T10-T20**	1.31	0.198	0.39	1.06	0.296	0.22
**T15-T20**	1.06	0.293	0.26	1.37	0.177	0.36
Behavior Rating Inventory of Executive Function (BRIEF)						
BRIEF - Emotion Regulation Index						
**T1-T5**	-1.43	0.162	-0.38	0.10	0.920	0.02
**T1-T10**	1.99	0.054	0.50	**3.65**	**<0.001**	0.70
**T1-T15**	**2.09**	**0.049**	0.54	**4.75**	**<0.0001**	1.02
**T1-T20**	1.66	0.114	0.42	**5.00**	**<0.0001**	1.01
**T5-T10**	**3.56**	**0.001**	0.91	**3.94**	**<0.001**	0.63
**T5- T15**	**3.29**	**0.002**	0.94	**4.77**	**<0.0001**	0.93
**T5-T20**	**2.64**	**0.014**	0.85	**4.94**	**<0.0001**	0.92
**T10-T15**	0.24	0.814	0.05	1.56	0.126	0.34
**T10-T20**	0.02	0.984	-0.10	1.72	0.093	0.34
**T15-T20**	-0.18	0.862	-0.16	0.35	0.731	0.02
BRIEF - Behavioral Regulation Index						
**T1-T5**	**-2.48**	**0.019**	-0.54	-0.39	0.696	0.06
**T1-T10**	1.24	0.223	-0.14	**3.49**	**0.001**	0.37
**T1-T15**	1.35	0.190	-0.42	**5.47**	**<0.0001**	0.44
**T1-T20**	1.02	0.320	-0.26	**5.34**	**<0.0001**	0.47
**T5-T10**	**3.72**	**<0.001**	0.49	**4.21**	**<0.001**	0.32
**T5- T15**	**3.37**	**0.002**	0.12	**5.89**	**<0.0001**	0.39
**T5-T20**	**2.70**	**0.012**	0.31	**5.67**	**<0.0001**	0.43
**T10-T15**	0.22	0.824	-0.34	**2.55**	**0.014**	0.07
**T10-T20**	0.01	0.996	-0.15	**2.23**	**0.031**	0.15
**T15-T20**	-0.18	0.857	0.18	-0.05	0.95	0.10
BRIEF – Emotional Control Index						
**T1-T5**	-0.90	0.373	-0.52	0.80	0.428	-0.07
**T1-T10**	1.30	0.203	0.32	**3.46**	**0.001**	0.66
**T1-T15**	2.12	0.045	0.38	**4.97**	**<0.0001**	1.11
**T1-T20**	1.35	0.191	0.44	**5.03**	**<0.0001**	1.05
**T5-T10**	2.36	0.024	0.93	**2.76**	**0.008**	0.67
**T5- T15**	2.93	0.006	1.02	**4.26**	**<0.001**	1.07
**T5-T20**	1.94	0.064	1.11	**4.32**	**<0.0001**	1.02
**T10-T15**	1.27	0.213	0.06	1.71	0.094	0.34
**T10-T20**	0.38	0.705	0.13	1.74	0.090	0.30
**T15-T20**	-0.60	0.549	-0.26	0.05	0.958	-0.04
BRIEF – Cognitive Regulation Index						
**T1-T5**	-1.55	0.129	-0.30	0.62	0.540	0.15
**T1-T10**	1.12	0.267	0.43	**3.21**	**0.003**	0.66
**T1-T15**	0.87	0.391	0.70	**2.87**	**0.007**	1.08
**T1-T20**	1.04	0.303	0.58	**3.97**	**<0.001**	0.95
**T5-T10**	2.79	0.008	0.75	**2.96**	**0.005**	0.47
**T5- T15**	2.06	0.045	0.99	**2.44**	**0.019**	0.87
**T5-T20**	1.99	0.052	0.91	**3.51**	**0.001**	0.76
**T10-T15**	-0.17	0.867	0.32	-0.10	0.923	0.47
**T10-T20**	0.23	0.820	0.16	1.07	0.290	0.37
**T15-T20**	0.42	0.679	-0.17	1.24	0.220	-0.07
BRIEF – General Executive Composite						
**T1-T5**	-1.40	0.172	0.49	0.40	0.688	0.06
**T1-T10**	1.68	0.099	0.57	**3.97**	**<0.001**	0.66
**T1-T15**	1.59	0.119	0.66	**5.43**	**<0.0001**	0.80
**T1-T20**	1.43	0.129	0.73	**5.24**	**<0.0001**	0.96
**T5-T10**	**3.31**	**0.002**	0.83	**4.12**	**<0.001**	0.57
**T5- T15**	**2.73**	**0.009**	0.89	**4.37**	**<0.0001**	0.70
**T5-T20**	**2.31**	**0.025**	0.04	**5.00**	**<0.0001**	0.84
**T10-T15**	0.14	0.893	0.15	1.09	0.281	0.12
**T10-T20**	0.17	0.865	0.14	1.80	0.079	0.24
**T15-T20**	0.09	0.928	-0.39	0.95	0.348	0.12
Flexibility Scale						
Flexibility Scale – Social subscale						
**T1-T5**	-0.65	0.521	-0.30	-0.68	0.504	0.28
**T1-T10**	**2.50**	**0.017**	0.60	1.52	0.134	1.42
**T1-T15**	1.02	0.321	0.41	0.31	0.755	0.96
**T1-T20**	1.12	0.280	0.57	**2.18**	**0.037**	1.32
**T5-T10**	**3.44**	**0.001**	0.95	**2.32**	**0.025**	0.99
**T5- T15**	1.58	0.125	0.74	0.84	0.406	0.57
**T5-T20**	1.63	0.118	0.92	**2.71**	**0.011**	0.93
**T10-T15**	-1.62	0.113	-0.21	-1.24	0.222	-0.47
**T10-T20**	-1.29	0.207	-0.06	0.81	0.422	0.02
**T15-T20**	0.14	0.890	0.16	**2.12**	**0.040**	0.44
Flexibility Scale – Transitions subscale						
**T1-T5**	**-2.77**	**0.009**	-0.02	**3.09**	**0.004**	-0.12
**T1-T10**	**2.02**	**0.049**	0.62	**7.06**	**<0.0001**	0.34
**T1-T15**	**2.25**	**0.033**	0.33	**4.26**	**<0.001**	-0.01
**T1-T20**	**2.45**	**0.023**	0.39	**5.16**	**<0.0001**	0.49
**T5-T10**	**4.91**	**<0.0001**	0.65	**5.05**	**<0.0001**	0.47
**T5- T15**	**4.53**	**<0.0001**	0.36	1.99	0.0539	0.14
**T5-T20**	**4.56**	**0.0001**	0.43	**2.84**	**0.009**	0.62
**T10-T15**	0.42	0.676	-0.30	**-2.52**	**0.015**	-0.45
**T10-T20**	0.63	0.535	-0.30	-1.39	0.173	0.19
**T15-T20**	0.31	0.755	0.03	0.99	0.329	0.62
Flexibility Scale – Generativity subscale						
**T1-T5**	**-3.21**	**0.003**	0.09	0.27	0.789	-0.14
**T1-T10**	-1.31	0.196	0.70	1.15	0.254	0.33
**T1-T15**	**-2.74**	**0.009**	0.58	1.57	0.122	0.29
**T1-T20**	-1.94	0.058	0.62	1.77	0.083	0.66
**T5-T10**	1.89	0.065	0.76	0.89	0.377	0.45
**T5-T15**	0.47	0.638	0.63	1.32	0.192	0.41
**T5-T20**	1.26	0.213	0.67	1.51	0.136	0.75
**T10-T15**	-1.42	0.162	-0.22	0.47	0.639	-0.05
**T10-T20**	-0.63	0.531	-0.13	0.64	0.526	0.31
**T15-T20**	0.79	0.434	0.08	0.15	0.882	0.38
Flexibility – Total Score						
***Total***						
**T1-T5**	-1.83	0.078	-0.38	1.92	0.063	0.02
**T1-T10**	1.89	0.066	0.50	**6.56**	**<0.0001**	0.70
**T1-T15**	1.17	0.250	0.54	**4.45**	**<0.0001**	1.02
**T1-T20**	1.70	0.982	0.42	**6.01**	**<0.0001**	1.01
**T5-T10**	**3.71**	**<0.001**	0.91	**5.56**	**<0.0001**	0.63
**T5- T15**	**2.89**	**0.006**	0.94	**3.08**	**0.004**	0.93
**T5-T20**	**3.42**	**0.002**	0.85	**4.59**	**<0.0001**	0.92
**T10-T15**	-0.73	0.469	0.05	-1.79	0.080	0.34
**T10-T20**	-0.19	0.854	-0.10	-0.04	0.967	0.34
**T15-T20**	0.54	0.591	-0.16	1.73	0.090	0.02
Clinical Global Impressions - Improvement						
**T5-T10**	**6.49**	**<.0001**	3.07	**6.35**	**<0.0001**	2.22
**T5- T15**	**5.47**	**<.0001**	2.60	**6.97**	**<0.0001**	2.66
**T5-T20**	**4.85**	**<.0001**	1.63	**7.55**	**<0.0001**	2.46
**T10-T15**	0.30	0.770	0.12	1.33	0.195	0.47
**T10-T20**	0.17	0.867	0.03	**2.11**	**0.044**	0.68
**T15-T20**	-0.08	0.939	-0.04	0.99	0.329	0.32

Contrasts in table represent the following: T1-T5, the 5-week control lead-in period; T1-T10, change from baseline to the end of the 5-week active intervention period; T1-T15, change from baseline to 5 week follow up assessments; T1-T20, change from baseline to 10 week follow-up assessments; T5-T10, change from beginning of intervention to the end of intervention; T5-T15, change from beginning of intervention to 5 week follow up assessments; T5-T20, change from beginning of intervention to 10 week follow-up assessments; T10-T15, change from the end of intervention to 5 week follow up assessments; T15-T20, change from 5 week follow up assessments to 10 week follow-up assessments. Bolded values indicate significant differences at the 0.05 level.

**Table 4:** Primary and secondary measure means by time point.

Measure	Younger	Older
	Mean (SE)	Mean (SE)
Emotion Dysregulation Inventory		
Reactivity subscale (Theta)		
**T1**	0.551 (0.181)	0.234 (0.220)
**T5**	0.515 (0.185)	0.234 (0.220)
**T10**	-0.030 (0.186)	-0.162 (0.221)
**T15**	-0.155 (0.192)	-0.080 (0.228)
**T20**	-0.216 (0.186)	-0.439 (0.225)
Dysphoria subscale (Theta)		
**T1**	0.075 (0.196)	0.159 (0.321)
**T5**	0.018 (0.199)	0.312 (0.321)
**T10**	-0.455 (0.200)	-0.259 (0.322)
**T15**	-0.328 (0.200)	-0.321 (0.329)
**T20**	-0.373 (0.200)	-0.552 (0.326)
Aberrant Behavior Checklist		
Irritability subscale		
**T1**	22.483(2.398)	18.746 (2.307)
**T5**	20.946 (2.424)	15.613 (2.307)
**T10**	14.929 (2.440)	12.101 (2.314)
**T15**	13.719 (2.450)	12.192 (2.361)
**T20**	11.714 (2.457)	10.301 (2.357)
Behavior Rating Inventory of Executive Function (BRIEF)		
Emotion Regulation Index		
**T1**	76.833 (2.463)	76.113 (2.540)
**T5**	79.266 (2.497)	75.979 (2.540)
**T10**	72.833 (2.463)	70.312 (2.549)
**T15**	72.417 (2.463)	67.919 (2.597)
**T20**	72.787 (2.729)	67.368 (2.604)
Behavioral Regulation Index		
**T1**	74.376 (2.040)	74.094 (2.403)
**T5**	78.811 (2.084)	74.560 (2.403)
**T10**	71.709 (2.040)	69.118 (2.410)
**T15**	71.293 (2.040)	65.609 (2.452)
**T20**	71.696 (2.381)	65.683 (2.458)
Emotional Control Index		
**T1**	72.116 (2.317)	71.931 (2.577)
**T5**	73.805 (2.358)	70.664 (2.577)
**T10**	69.033 (2.317)	66.107 (2.588)
**T15**	66.533 (2.317)	63.096 (2.646)
**T20**	67.971 (2.683)	63.000 (2.645)
Cognitive Regulation Index		
**T1**	70.155 (2.390)	69.173 (3.200)
**T5**	73.027 (2.428)	68.506 (3.200)
**T10**	67.405 (2.390)	65.026 (3.204)
**T15**	67.738 (2.390)	65.148 (3.231)
**T20**	66.754 (2.730)	63.593 (3.234)
General Executive Composite		
**T1**	75.581 (2.124)	74.343 (2.864)
**T5**	78.009 (2.162)	73.877 (2.864)
**T10**	71.747 (2.124)	68.635 (2.870)
**T15**	71.747 (2.124)	67.156 (2.905)
**T20**	71.297 (2.451)	65.822 (2.912)
Flexibility Scale		
Social subscale		
**T1**	8.596 (0.913)	7.696 (0.964)
**T5**	8.968 (0.920)	8.096 (0.964)
**T10**	6.864 (0.923)	6.591 (0.968)
**T15**	7.857 (0.924)	7.447 (0.994)
**T20**	7.772 (0.924)	5.972 (0.985)
Transitions subscale		
**T1**	10.983 (1.145)	12.911 (1.164)
**T5**	13.047 (1.154)	1.111 (1.164)
**T10**	9.119 (1.159)	7.888 (1.168)
**T15**	8.782 (1.160)	9.604 (1.190)
**T20**	8.530 (1.161)	8.929 (1.182)
Generativity subscale		
**T1**	7.231(0.663)	8.753 (0.657)
**T5**	8.921(0.671)	8.620 (0.657)
**T10**	7.921(0.671)	8.168 (0.661)
**T15**	8.671(0.671)	7.914 (0.681)
**T20**	8.255 (0.671)	7.832 (0.671)
Total subscale		
**T1**	43.898 (2.887)	44.984(2.993)
**T5**	48.554 (2.930)	42.184 (2.993)
**T10**	38.851 (2.933)	33.291 (3.001)
**T15**	40.766 (2.933)	36.335 (3.054)
**T20**	39.349 (2.933)	33.369 (3.034)
Clinical Global Impressions - Improvement		
**T1**	Not recorded	
**T5**	4.412 (0.297)	4.181 (0.246)
**T10**	2.578 (0.297)	2.584 (0.255)
**T15**	2.495 (0.297)	2.228 (0.263)
**T20**	2.518 (0.306)	1.944 (0.271)

Timepoints above represent the following: T1 = beginning of 5-week control lead-in period, T5 = beginning of 5-week active intervention period, T10 = end of 5-week active intervention period, T15 = follow up assessments at 5-weeks, and T20 = follow up assessments at 10-weeks.

#### Emotion dysregulation inventory reactivity and dysphoria

**Figure 2:**
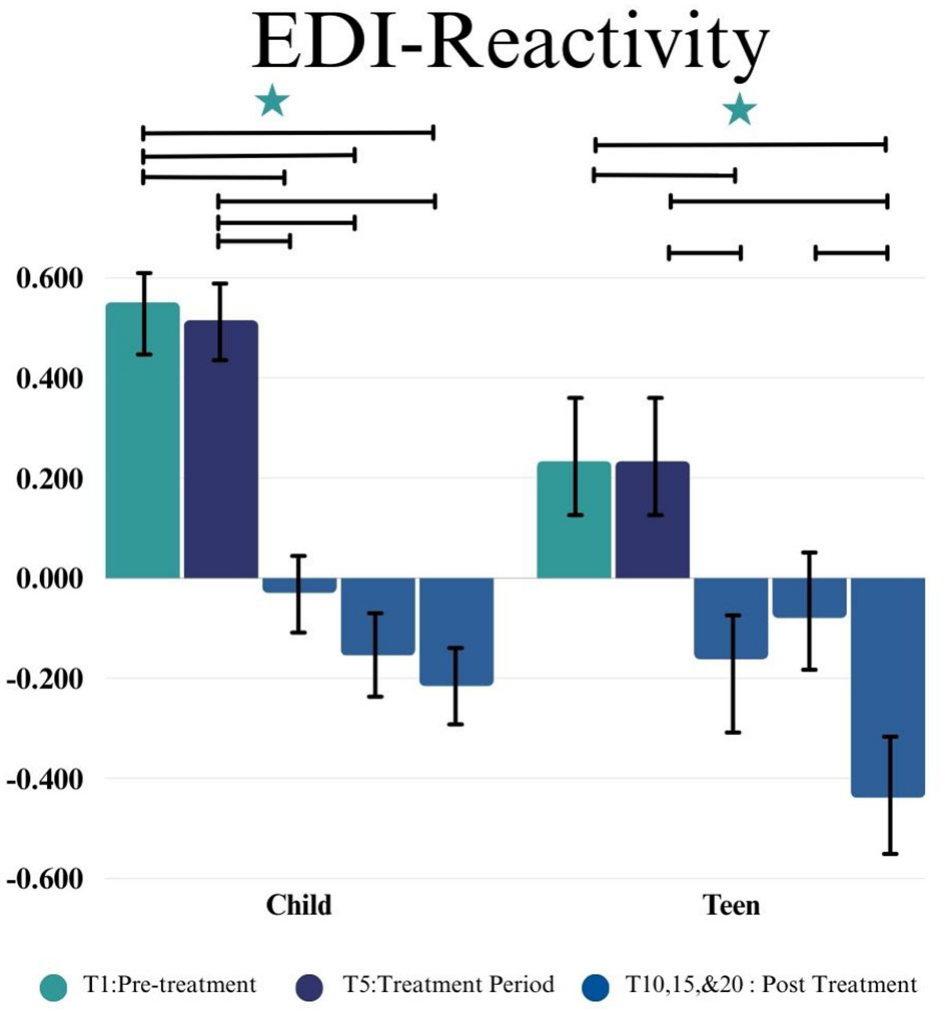
This figure depicts the significant difference on EDI-R across time points (T1, T5, T10, T15, and T20) in Child and Teen groups. Stars depict significance at the 0.05 level.

In the Child group for the EDI-R, a statistically significant difference was observed by time point (*F*(4,40.67) = 3.45, *p* = 0.016). No significant change in scores was observed during the 5-week control lead-in period (*p* = 0.846, *d* = 0.05). Improvement on EDI-R from the start of the intervention (T5) to post-intervention (T10) was observed (*p* = 0.009, *d* = 0.86). Significant improvement on the EDI-R also was found from intervention start to both 5 weeks (T15) (*p* = 0.007, *d* = 0.99) and at the 10-week follow-up (T20) for the child group (*p* = 0.005, *d* = 1.13) (**Figure 2**).

**Figure 3:**
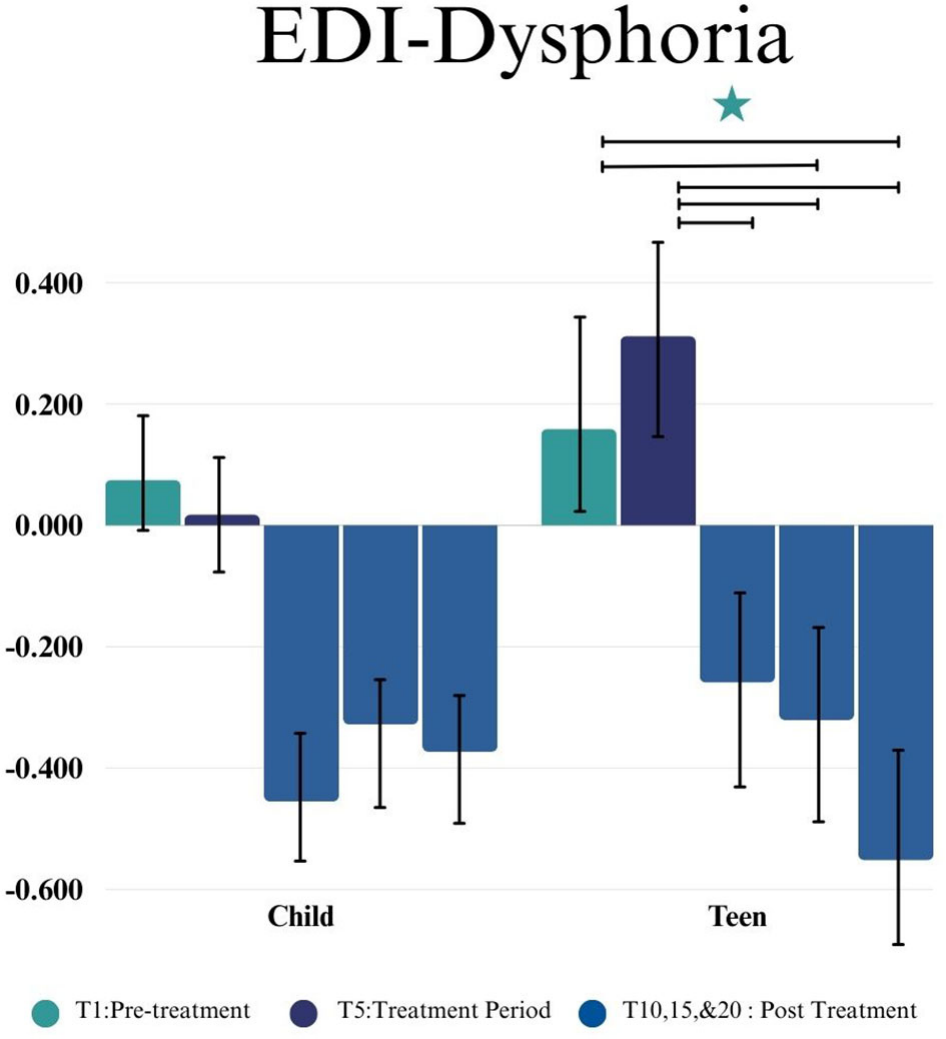
This figure depicts the significant difference on EDI-D across time points (T1, T5, T10, T15, and T20) in Child and Teen groups. Stars depict significance at the 0.05 level.

In the Teen group, a statistically significant difference was observed in EDI-R by time point (*F*(4,35.24) = 5.07, *p =* 0.0025). No significant change in score during the 5-week control lead-in period (T1-T5) (*p* = 0.950, *d* = 0.01) was observed. Improvement on the EDI-R from intervention start (T5) to post-intervention (T10) was observed (*p* = 0.023, *d* = 0.45). There was not a significant difference from intervention start to 5 Weeks post-intervention (T15)(*p* = 0.092, *d* = 0.38) but there was a significant reduction in EDI-R scores between intervention start and 10 weeks post-intervention (T20)(*p* < 0.001, *d*= 0.84) (as shown in **Figure 3**).

For the Child group there were not significant differences in EDI-D (*F*(4,33.01) = 4.17, *p =* 0.0077) between any of the time points. However, in the Teen group (*F*(4,41.46) = 2.05, *p =* 0.1004) there were significant differences between the intervention start (T5) and post-intervention (T10) (*p* = 0.004, *d* = 0.78), 5 weeks post-intervention (T15) (*p* = 0.008, *d* = 0.24), and 10 weeks post-intervention (T20) (*p* = 0.001, *d* = 0.55), such that teens experienced a decrease in dysphoria subsequent to completing the group. There was no significant change in score between the lead-in period (T1-T5) for the teens (*p* = 0.385, *d* = 0.47) (as shown in **Figure 3**).

#### ABC irritability subscale

**Figure 4:**
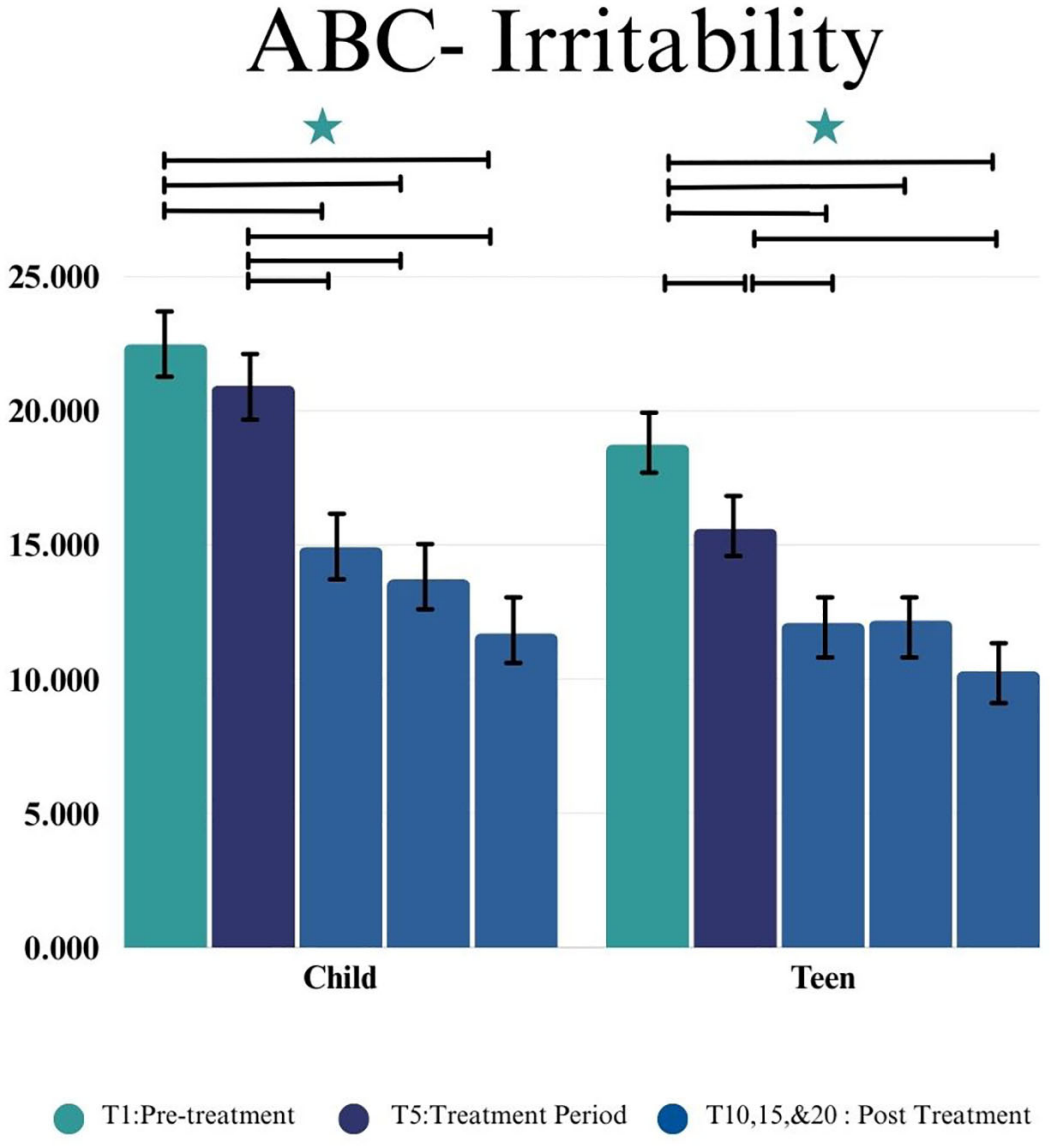
This figure depicts the significant difference on ABC Irritability subscale across time points (T1, T5, T10, T15, and T20) in Child and Teen groups. Stars depict significance at the 0.05 level.

For the Child group we found a statistically significant difference in the ABC Irritability subscale by time point (*F*(4,34.97) = 4.22, *p* = 0.007). No change in scores during the 5-week control lead-in period (*p* = 0.374, *d* = 0.28) were observed. Improvement in the ABC Irritability subscale from intervention start (T5) to post-intervention (T10) was observed (*p* = 0.003, *d* = 0.70), as was significant reduction in irritability from intervention start to both the 5 week (T15) (*p* = 0.006, *d* = 0.85) and 10 week follow-up (T20) (*p*=0.003, *d* = 1.20) (shown in **Figure 4**).

For the Teen group we found a statistically significant difference in the ABC Irritability subscale by time point (*F*(4,35.45) = 5.90, *p =* 0.001). Significant improvement, or change in score, during the 5-week control lead-in period (T1-T5) was observed (*p* = 0.009, *d* = 0.47). There was also improvement on the ABC Irritability subscale from intervention start (T5) to post-intervention (T10) (*p* = 0.009, *d* = 0.41). There was not a significant difference from intervention start to 5 Weeks post-intervention (T15) (*p* = 0.051, *d* = 0.36) but there was a significant reduction in irritability observed between intervention start and 10 weeks post-intervention (T20) (*p* =0.01, *d* = 0.79) (as shown in **Figure 4**).

### Secondary outcome measures

#### BRIEF

For the Child group, statistically significant changes were observed by time point on the BRIEF ERI (*F*(4, 28.68) = 3.70, *p*=0.015) and BRI (*F*(4, 31.07) = 4.19, *p*=0.0079), and the GEC (*F*(4, 38.7) = 3.01, *p* = 0.0294), such that children showed fewer emotion regulation, cognitive regulation challenges after completing the group, and better general executive functioning skills. No significant changes were observed by timepoint in the ECI (*F*(4,31.18) = 2.20, *p =* 0.0918) or CRI in the Child group (*F*(4,38.46) = 2.30, *p =* 0.0759).

For the Teen group, statistically significant changes were demonstrated by time point on the ERI (*F*(4, 38.57) = 9.69, *p* < 0.0001), the BRI (*F*(4, 37.69) = 12.34, *p* < 0.0001), the ECI (*F*(4,39.24) = 10.27, *p <* 0.0001), the CRI (*F*(4, 37.27) = 5.17, *p* = 0.002), and the GEC (*F*(4, 37.95) = 9.42, *p* < 0.0001). This indicates that teens showed fewer emotion regulation, behavior regulation, emotional control, and cognitive regulation challenges after completing the group, and better general executive functioning skills.

**Figure 5:**
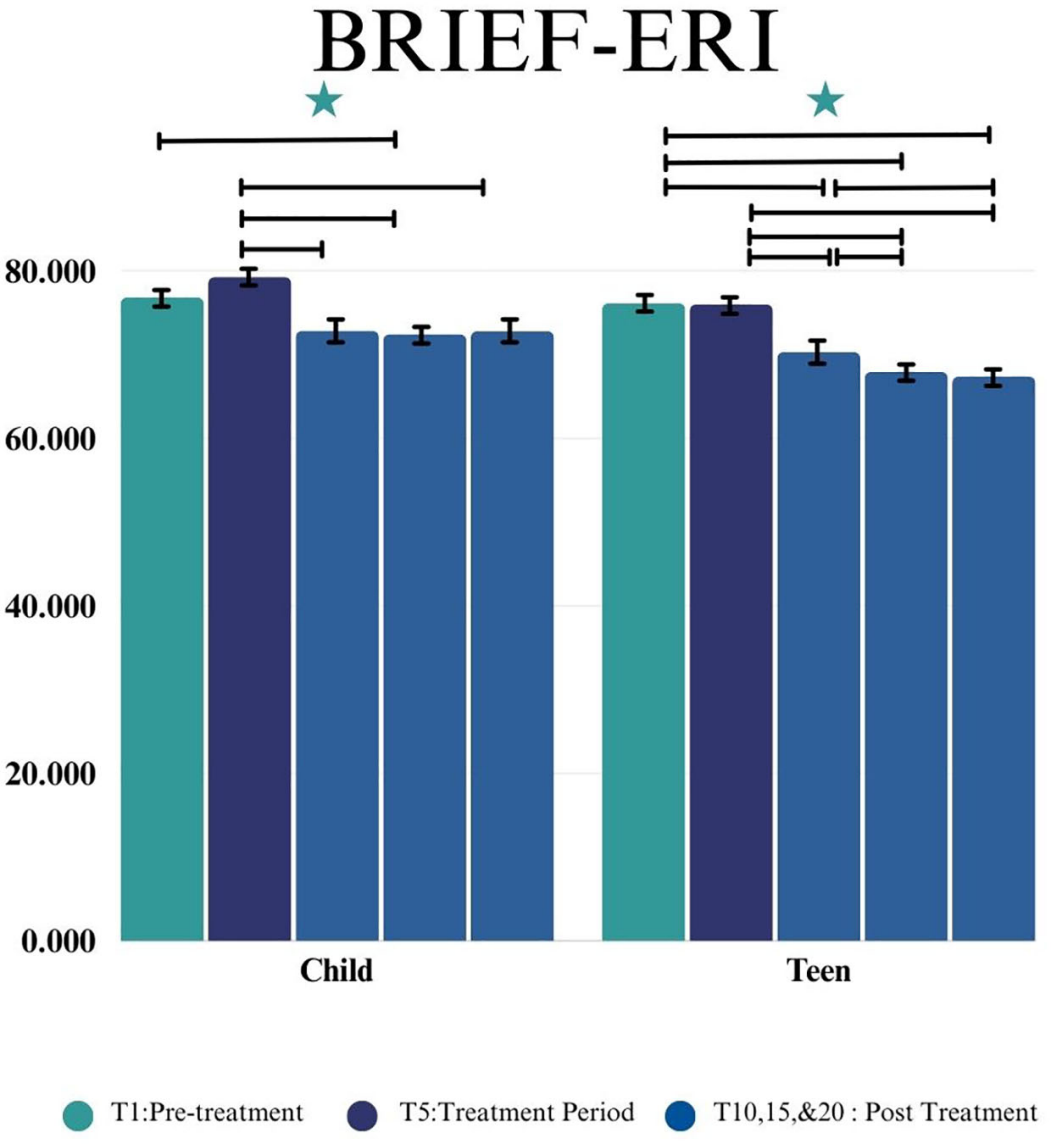
This figure depicts the significant difference on BRIEF- Emotion Regulation Index (ERI) across time points (T1, T5, T10, T15, and T20) in Child and Teen groups. Stars depict significance at the 0.05 level.

Further analyses are presented in **Table 3** and change post-intervention are shown in **Figure 5**.

#### Flexibility scale

**Figure 6:**
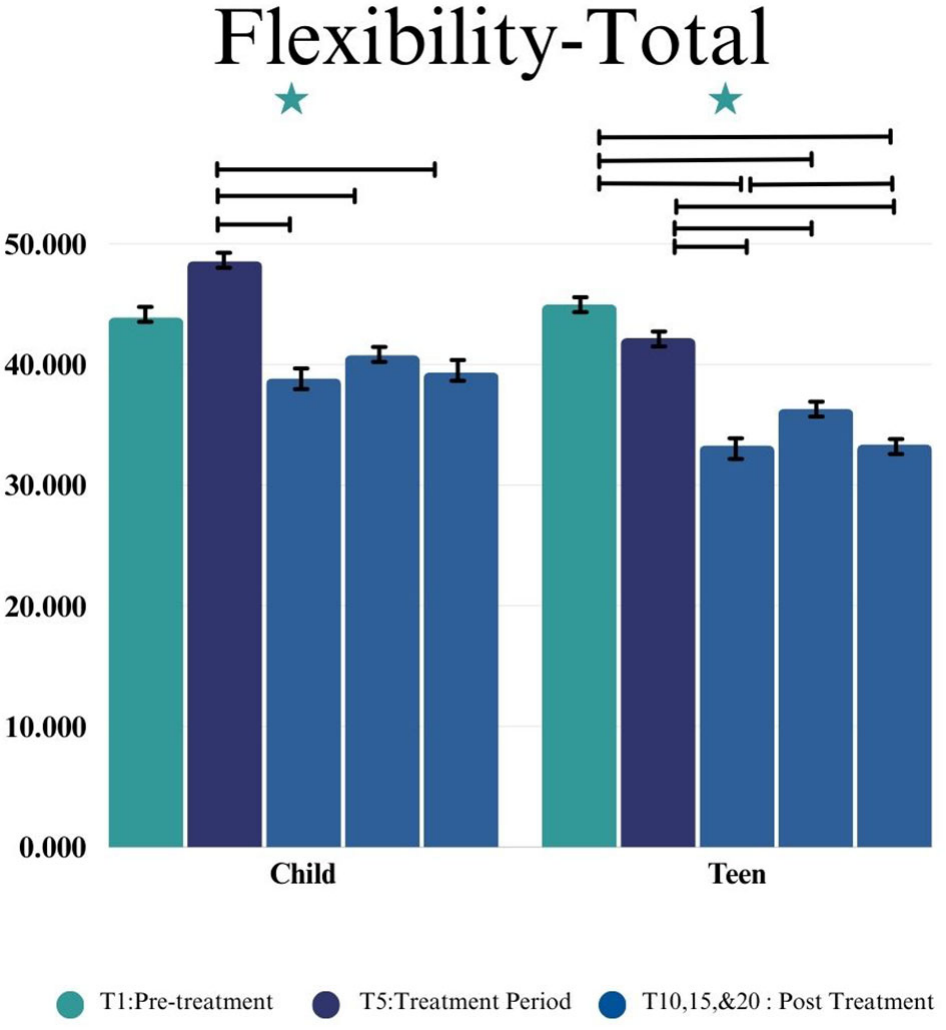
This figure depicts the significant difference on Flexibility-Total across time points (T1, T5, T10, T15, and T20) in Child and Teen groups. Stars depict significance at the 0.05 level.

In the Child group, a statistically significant change by time point was observed on the FS Total point (*F*(4,33.51) = 4.34, *p* = 0.0062), shown in **Figure 6**. Statistically significant changes by time point were also found on the following subscales: Social Flexibility (*F*(4,29.00) = 2.97, *p* = 0.0357), Transitions/Change (*F*(4,33.32) = 7.76, *p* = 0.002), and Generativity (*F*(4,44.17) = 3.18, *p* = 0.0222), such that children showed better social flexibility, better adaptability to transitions and changes, and better ability to generate ideas after completing the group.

In the Teen group, a statistically significant change in the FS Total score was observed by time point (*F*(4,37.81) = 14.53, *p=* 0.0001), shown in **Figure 6**). Significant changes by time point were also observed Social Flexibility (*F*(4,38.86) = 2.84, *p* = 0.0372) and Transitions/Change (*F*(4,34.46) = 13.41, *p* < 0.0001) subscales, such that teens demonstrated better social flexibility and better adaptability to transitions after completing the group. However, in the Teen group the Generativity subscale presented no significant changes (*F*(4,49.99) = 1.23, *p* = 0.3111). Further analyses are presented in **Table 3**.

#### Clinical global impression – improvement

On the CGI-I for the Child group, statistically significant improvement was observed by time point (*F*(3,28.62) = 15.54, *p* < 0.0001). On the CGI-I for the Teen group there was significant improvement by time point (*F*(3,22.89) = 24.60, *p*<0.0001). The most significant improvement was observed between the beginning to the end of the intervention T5 and T10, as well as with the two follow-up time points (T5 and T15, and T5 and T20) for both groups. Further analyses are presented in **Table 3**.

## Discussion

For families and individuals with ASD or other psychiatric conditions, telehealth options for interventions can increase ease and access (8). When RT was originally tested in person, it demonstrated significant improvement in emotion regulation based on parent report and reduced psychiatric hospitalization rates (11). Overall, children and teens participating in the RT telehealth format demonstrated similar significant improvements in their emotion regulation and reductions in irritability.

Specifically, in both age groups we observed statistically significant improvement on both our primary measures of ED, suggesting RT delivered through telehealth showed preliminary efficacy at reducing reactivity and irritability in autistic youth. Notably, for both of these primary outcome measures there was stability in the lead in period which suggests the improvement is in response to the intervention and not a result of the passage of time. Also, the continued improvement at the 5- and 10-week follow-up suggests continued benefit, or maintenance of positive effects, from the intervention across time. Additionally, the Teen group showed significant improvement on the dysphoria scale of the EDI, a finding that was not observed in the Child group. One potential explanation for this difference is the already lower EDI-D ratings for the Child group compared to the Teen group. Additionally, because the two groups differed slightly in material, the Teen intervention may have had a bigger impact on emotional dysphoria than the Child intervention. Both age groups also showed improvements on the CGI-I suggesting not only improvement based on parent report but also clinician ratings.

We also saw significant changes in executive functioning as measured by the BRIEF for both age groups. For the Child group, significant improvement in emotion regulation, behavior regulation, and general executive skills were reported immediately post intervention and at the 10-week follow-up. Similar results also occurred in the Teen group, with additional improvements in cognitive regulation and emotional control noted both immediately after the completion of the intervention as well as weeks after completing the group. In terms of flexibility, parents of the child group noted significant improvement overall and on two subscales. While there was a significant difference in the lead-in period on the Transitions subscale this difference was negative suggesting a worsening of transitional flexibility prior to intervention, making this improvement more remarkable. For teens, caregivers noted improvements in all subscales aside from generativity. Together, this suggests that telehealth RT may not only help reduce core ED symptoms, but also broader aspects known to be related to ED.

We also examined differences between the two groups. Across several measures, teens showed greater improvements compared to children. These improvements were shown on the dysphoria subscale of the EDI and on the Emotional Control and Cognitive Regulation subscales of the BRIEF. These results should be viewed in light of the in-person intervention results, in which teens did not show these same gains. Evidence suggests that resistance to attending sessions is particularly tenuous for adolescents, which may impede participation in intervention (18). Thus, our findings of good attendance rates and significant emotion regulation improvement in this population indicate that RT may be especially beneficial for adolescents when it is delivered through telehealth.

### Limitations

As discussed in the original study (11), the primary limitation of the current study is the within-subject study design as opposed to a RCT. Although an RCT would be ideal to test the effectiveness of an intervention this study design is an important step to evaluate initial efficacy of this intervention especially in the telehealth format. Telehealth interventions are an emerging area for autistic youths, and thus this examination still provides an important contribution to the field (19). Another previously discussed limitation is the primary use of caregiver report to measure outcomes. Future research could include child report as well as other quality-of-life measures to get a more holistic evaluation of intervention’s effectiveness. The findings should also be considered in light of global events occurring at the time, namely the COVID-19 pandemic. Contemporaneous research suggests that autistic youths experienced even greater psychological distress as a result of the pandemic (6), and therefore the availability of an intervention may have been particularly helpful for these participants. The rapid adjustment to telehealth necessitated a number of changes to our typical protocol, including relaxed inclusion criteria. Further, children were experiencing a global crisis that may have had an impact on their emotion regulation skills regardless of an intervention. An additional limitation includes the lack of diversity in the study sample. Although national recruitment was completed, the sample was primarily non-Hispanic, white families with higher socioeconomic status and education levels. Future studies should focus on more diverse recruitment as well as facilitators and barriers to participation for diverse families and our team is currently conducting a trial with a focus on diverse recruitment to Regulating Together (ClinicalTrials.gov ID NCT05803369). Finally, again because of the nature of the COVID-19 pandemic, typical phenotyping measures were not available, including cognitive scores and autism diagnostic measures. It will be helpful for future studies to provide thorough phenotyping in order to better understand response to the intervention.

## Conclusion

Emotion regulation research is critical due to its perceived connection between behavioral concerns, social adaptability and overall well-being in individuals with ASD. Additionally, creating high-quality, effective telehealth interventions for autistic youths and their families is necessary to ensure mental health care is equitable and accessible. The results from this study support our hypotheses that the telehealth format of Regulating Together would improve emotion regulation and overall function as observed in both age groups, with a particular benefit noted for adolescents. These findings are very promising for individuals and families with ASD and ED especially those that require telehealth settings in order to receive intervention, thereby potentially improving access to care, and require further examination with a larger, more diverse sample.

## Data Availability

The raw data supporting the conclusions of this article will be made available by the authors, without undue reservation.
